# Genomic instability in complicated and uncomplicated Egyptian schistosomiasis *haematobium* patients

**DOI:** 10.1186/s13039-014-0104-5

**Published:** 2015-01-22

**Authors:** Amany A Abd El-Aal, Ibrahim R Bayoumy, Maha M A Basyoni, Asmaa A Abd El-Aal, Ashraf M Emran, Magda S Abd El-Tawab, Manal A Badawi, Rabab M Zalat, Tarek M Diab

**Affiliations:** Parasitology Department, Faculty of Medicine, Cairo University, Cairo, Egypt; Parasitology Department, Theodor Bilharz Research Institute, Giza, Egypt; Clinical Pathology Department, Faculty of Medicine, Cairo University, Cairo, Egypt; Urosurgery Department, Faculty of Medicine, Cairo University, Cairo, Egypt; Pathology Department, National Research Center, Giza, Egypt

**Keywords:** Schistosomiasis *haematobium*, Chromosomal abnormalities, Morphocytometry, FISH, Karyotyping

## Abstract

**Background:**

Exploration of genetic changes during active *Schistosoma* infection is important for anticipation and prevention of chronic sequelae. This study aimed to explore the genomic instability in chromosomal and cellular kinetics in Egyptians suffering from uncomplicated active schistosomiasis *haematobium* infection in addition to chronic schistosomiasis *haematobium* cases complicated by bilharzial-associated bladder cancer (BAC).

**Results:**

This study was conducted on 46 schistosomiasis *haemotobium* cases, 22 were active (Viable *S. haematobium* eggs in urine samples as detected by microscopy) and 24 were chronic complicated with bladder cancer. Three cytogenetic techniques were applied; the first was quantitative nuclear-morphocytometry by means of which the Feulgen-stained nuclei were analyzed for parameters including shape, size, integrated optical-density and nuclear area. The second was Fluorescent In-Situ Hybridization (FISH) for specific p53gene-locus of chromosome 17 and the third technique was karyotyping.

Concerning chronic complicated cases, the mean ± SD of DNA-content in urinary bladder tissue sections was 3.18 ± 0.65. Five samples (20.83%) of bladder tissue sections of chronic complicated cases showed diploid nuclei, 6 urinary bladder tissue samples (25%) were tetraploid, while 13 bladder samples (54.16%) were aneuploid. Epithelial cells of urine samples demonstrated aneuploidy (mean ± SD = 3.74 ± 0.36).Nuclear contents showed high proliferative DNA index in all urinary epithelial cells. In the acute uncomplicated group, nuclear-DNA of urinary epithelial cells was found diploid with mean nuclear-DNA content of 2.2 ± 0.16SD. Half of these diploid smears had a high proliferation index. The difference between nuclear DNA-contents in acute and chronic cases was significant (P = 0.0001). FISH technique for specific p53gene-locus and karyotyping were done on urinary bladder tissue specimens and peripheral blood monocytes of 8 chronic cases respectively. Three samples (37.5%) with invasive BAC had a deletion of the p53 gene. Karyotyping showed three cases out of the 8 chronic schistosomiasis *haematobium* patients with chromosomal fragmentations.

**Conclusions:**

DNA morphometry was valuable in detection of gross genetic changes in urothelial tissues. It is an important prognostic factor in established schistosomiasis *haematobium* induced bladder malignancy. It has the great advantage of being applicable on urine cells making it suitable for the prediction of a tendency towards genetic instability in active schistosomiasis *haematobium* patients.

## Background

Schistosomiasis is a parasitic disease that dates back to ancient Egyptians who were among the first to contract the disease The chief symptom, haematuria, was mentioned in the Egyptian papyri (1500–1800 B.C.). Schistosomiasis, also known as bilharzia, is caused by trematodes from the genus *Schistosoma*. Transmission occurs in stagnant or slow-moving fresh water where infected *Bulinus* snails live. Transmission rates to populations that have frequent exposure to water [1].

Urogenital schistosomiasis results when adult female *S. haematobium* worm pairs living in the veins draining key pelvic organs, including the bladder, uterus, and cervix, release terminal-spine eggs that penetrate the tissues and are excreted in the urine to allow propagation of the parasite life cycle. Schistosomiasis is due to immunologic reactions to *Schistosoma* eggs trapped in tissues. Antigens released from the egg stimulate a granulomatous reaction involving T cells, macrophages, and eosinophils that results in clinical disease. This infection has a significant and specific impact on the urino-genital system and has a strong association with bladder cancer, leading to severe and chronic morbidity [2].

Urinary schistosomiasis constitutes a major global health burden due to its devastating complications including chronic bacterial urinary tract infections and dysfunction caused by the parasite. The bladder may also develop tubercles, polyps, ulcers, sandy patches, cystitis cystica, and/or leukoplakia which may progress to carcinoma of the bladder that are visible upon endoscopic examination [1]. In addition, *S. haematobium* has a high prevalence in particular foci in which it is endemic including over 50 countries in Africa and the Middle East, while it occupies certain foci of Asia. Its causative agent, *Schistosoma haematobium*, has acquired the title of the neglected schistosome explained by relying solely on data concerning the much more extensively studied intestinal form of schistosomiasis caused by *S. mansoni*. The current lack of genome and transcriptome information for *S. haematobium* is directly hindering further targeted research. There is a wide spectrum of chronic sequelae of urinary schistosomiasis ranging from chronic cystitis to the development of carcinoma of the bladder [3]. Bladder cancer accounts for 30.8% of the total cancer incidence among Egyptians and is ranked first among all types of cancer recorded in Egyptian males and second only to breast cancer in females [4]. Healthy cellular growth and multiplication of urothelial tissues is essential to guard against urothelial neoplastic changes. Therefore, disturbances in cellular kinetics and cell cycle dynamics play a pivotal role in the genesis of chromosomal abnormality and consequently, the development of malignancy [5].

Invasive and/or expensive procedures for diagnosing *S. haematobium* usually interfere with early diagnosis of possible malignant transformation of urothelial tissues. Applying a non invasive, affordable technique will certainly minimize the number of complicated bilharzial cases in our country being supported by patients’ convenience and compliance.

DNA specific dyes, such as Feulgen stain and ploidy analysis interprets chromosomal content as a whole, while karyotyping allows a closer look at each individually studied peripheral blood mononuclear cell chromosome. In addition, Fluorescence in situ hybridization (FISH) has been used to take a closer look, than other chromosomal studying techniques, at chromosomal abnormalities by detecting changes in a single locus or in multiple gene loci [6]. P53 gene, located on the short arm of chromosome 17, is one of the most intensively investigated tumor suppressor genes in human cancer including cancer bladder [7]; it encodes an essential protein involved in the growth and regulation of cell proliferation and DNA damage control response by promoting apoptosis [8]. Nitric oxide produced by the inflammatory response provoked by schistosomal eggs was discovered to cause p53 gene mutation [9]. For early prediction of neoplastic transformation through investigating abnormal cytogenesis, the present study aims at exploring the chromosomal and cytokinetic genomic instability in Egyptian patients suffering from *S. haematobium* infection through detection of chromosomal abnormalities in their urothelium using quantitative nuclear densitometry of urothelial cells. In addition, alteration in p53 gene using FISH technique and locus specific probe could be detected. On the other hand, chromosomal abnormalities in peripheral blood lymphocytes of patients suffering from schistosomisis *haematobium* infections were studied by karyotyping.

## Results

### Demographic data

The present study included 43 (93.48%) male patients and 3 (6.52%) female patients. These 3 female patients were all among the chronic patients group. The difference in gender distribution was found to be statistically significant (P <0.0001) (Table 1). The ages among the active, uncomplicated infection group ranged from 6 to 25 years. The ages of the chronic age group ranged from 38 years to 84 years (P = 0.0001). As regards the geographical distribution, in active group, only one patient came from Menya (Upper Egypt) (2.17%) while the remaining patients were from rural or urban regions (Lower Egypt). In chronic group, 14 patients were from Lower Egypt and 10 cases were from Upper Egypt. A statistically significant difference between the distribution of infection in Upper and Lower Egypt in both active and chronic infection groups was found (P = 0.0001) (Table 2).

**Table 1:** **Sex distribution among study groups**

	**Male**		**Female**		**Total**
	**No**	**%**	**No**	**%**	
Active *S. haematobium* infection group (n = 22)	22	100	0	00.0	22
Chronic *S. haematobium* infection group (n = 24)	21	87.50	3	12.50	24
Total (n = 46)	43	93.48	3	6.52	46

**Table 2:** **Geographical distribution among active and chronic infection groups**

***Residence***	**Active infection group**		**Chronic infection group**		**Total**	
	**No**	**%**	**No**	**%**	**No**	**%**
**Giza (rural)**	9	19.56%	7	15.22	16	34.78
**Giza (urban)**	6	13.04%	0	00.00	6	13.04
**Cairo (urban)**	6	13.04%	7	15.22	13	28.26
**Menya**	1	2.17%	3	6.52	4	8.7
**Aswan**	0	00.00%	2	4.35	2	4.35
**Souhag**	0	00.00%	2	4.35	2	4.35
**Fayoum**	0	00.00%	3	6.52	3	6.52
**Total**	22	47.83%	24	52.17	46	100.00%

### Cytogenetic diagnosis

#### DNA ploidy analysis

17 patients of the chronic infection group (n = 24) suffered from squamous cell carcinoma (SCC) associated with bilharziasis (70.83%), while 7 (29.17%)patients suffered from bilharziasis associated transitional cell carcinoma(TCC) associated with bilharziasis (29.17%) as evidenced by their pathology reports (Table 3).

**Table 3:** **Histopathological type of BAC among the chronic infection group (n = 24)**

	**SCC**	**TCC**
**Number**	17	7
**Percent**	70.83%	29.17%

**Figure 1:**
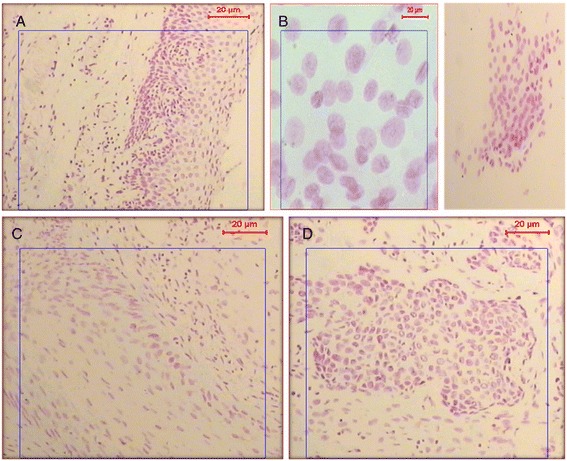
**Feulgen stained nuclei of epithelial cells. (A)** Nuclei of epithelial cells showing a normal diploid nuclear content. **(B)** Feulgen stained nuclei of malignant epithelial cells found in the urine smear of a chronic urinary bilharziasis patient. The nuclei show aneuploid DNA content with high proliferation pattern with 100× and 400× magnification for A&B respectively. **(C & D)** Feulgen-stained nuclei of epithelial cells from tissue sections of a biopsy sample showing bilharzial associated carcinoma.

The mean ± SD of DNA content in tissue sections of chronic complicated patients was 3.18 ± 0.65 .Five samples (20.83%) showed diploid nuclei, 6 samples (25%) were tetraploid, while 13 samples (54.16%) were aneuploid. They all showed high proliferative index (Figure 1). A comparative group composed of 4 non-bilharzia associated bladder carcinoma (BAC) patients was studied by Feulgen stained ploidy analysis. These samples included 2 samples with diploid nuclei and two samples with tetraploid nuclei with high proliferative index. The mean ± SD of their DNA content was 2.54 ± 0.3. No significant statistical difference was found between the two groups (P = 0.068) (Table 4). In urine samples, nuclei of epithelial cells of chronic patients demonstrated aneuploidy with a high proliferative index with mean ± SD of DNA content 3.74 ± 0.36.

**Table 4:** **Comparison between DNA content of BAC versus non-BAC**

**Type of cancer**	**Mean DNA content**	**±SD**
**BAC**	3.18	±0.65
**Non-BAC**	2.54	±0.3

**Figure 2:**
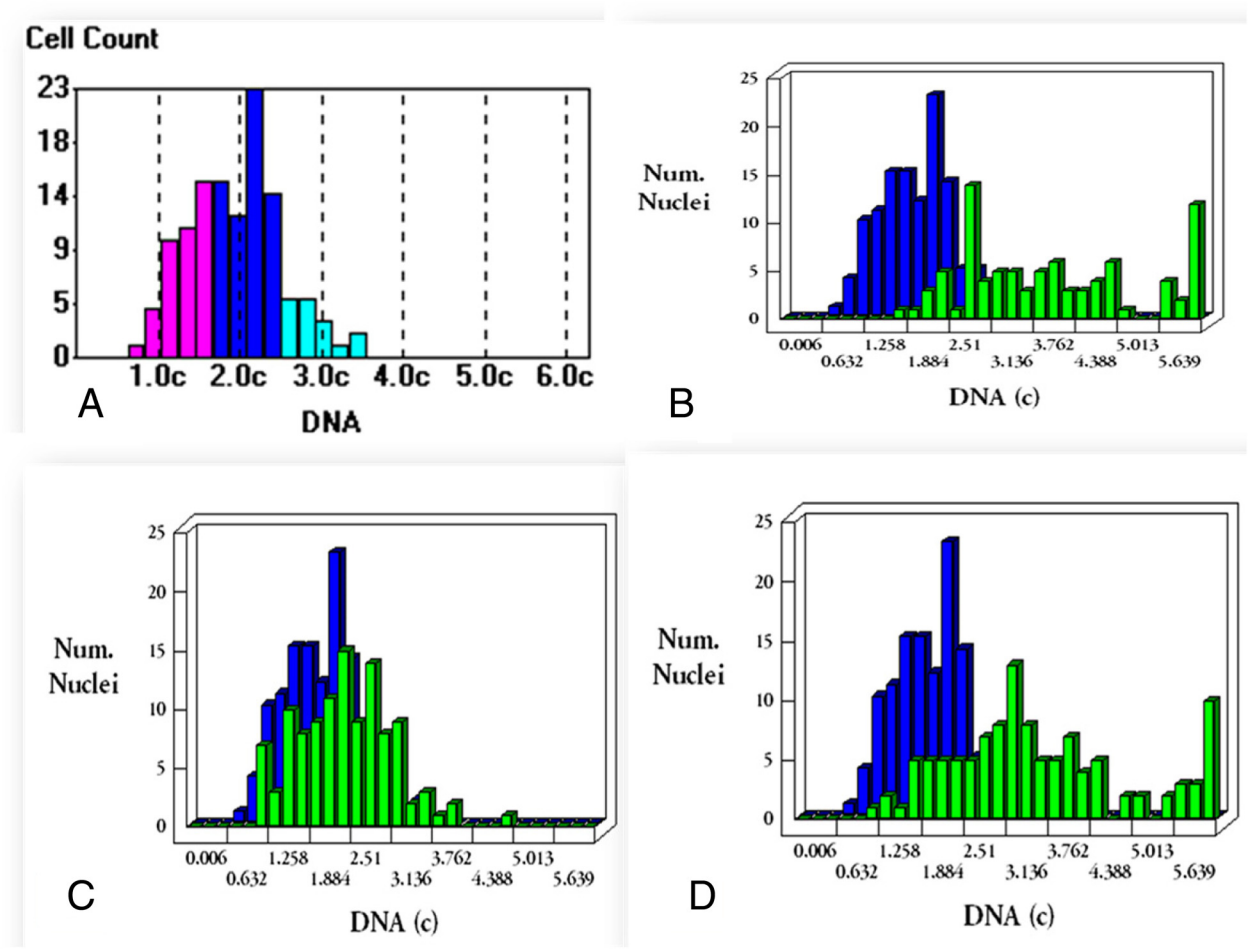
**Combo-blot showing the histograms of Feulgen stained samples. (A)** Relative distribution of DNA ploidy pattern among cells of the reference normal sample. **(B)** Urine smear from a chronic *S. haematobium* patient showing aneuploidy with high proliferation (green) blotted against the histogram of the reference sample (blue). **(C)** Epithelial cells of bilharzial induced bladder carcinoma revealing diploidy with a high proliferation pattern (green) blotted against the histogram of the reference sample (blue) **(D)** Feulgen stained tissue section from a chronic *S. haematobium* patient with bilharzial induced carcinoma showing aneuploidy with a high proliferation pattern (green) blotted against the histogram of the reference sample (blue).

While, in the acute uncomplicated group, nuclear DNA of urinary epithelial cells was found to be diploid with mean nuclear DNA content of 2.2 ± 0.16. Half of these diploid smears were found to have a high proliferation index. The difference between the mean DNA content of epithelial cell nuclei in acute and chronic cases was found to be statistically significant (P = 0.0001) (Table 5). The nuclear DNA contents of the cells were measured relative to the nuclear content of normal diploid nuclei from a reference control registered on the image analyzing soft-ware system using the Leica Qwin 500 Image Analyzer (LEICA Imaging Systems Ltd, Cambridge, England,). The mean DNA content of these reference nuclei (1.96) was taken as a reference value for the mean DNA content of the present study group (Figure 2, Combo-blot showing the histograms).

**Table 5:** **Comparison between mean DNA content of epithelial cells in positively stained urine smears of active and chronic patients**

**Study group**	**Percent of patients with positive Feulgen staining of urine (100%) = 46**	**DNA content** ***Mean*** **±** ***SD***
**Active infection group**	8.70%	2.2 ± 0.16
**Chronic infection group**	10.7%	3.74 ± 0.36

#### Detection of p53 deletion by FISH technique

**Figure 3:**
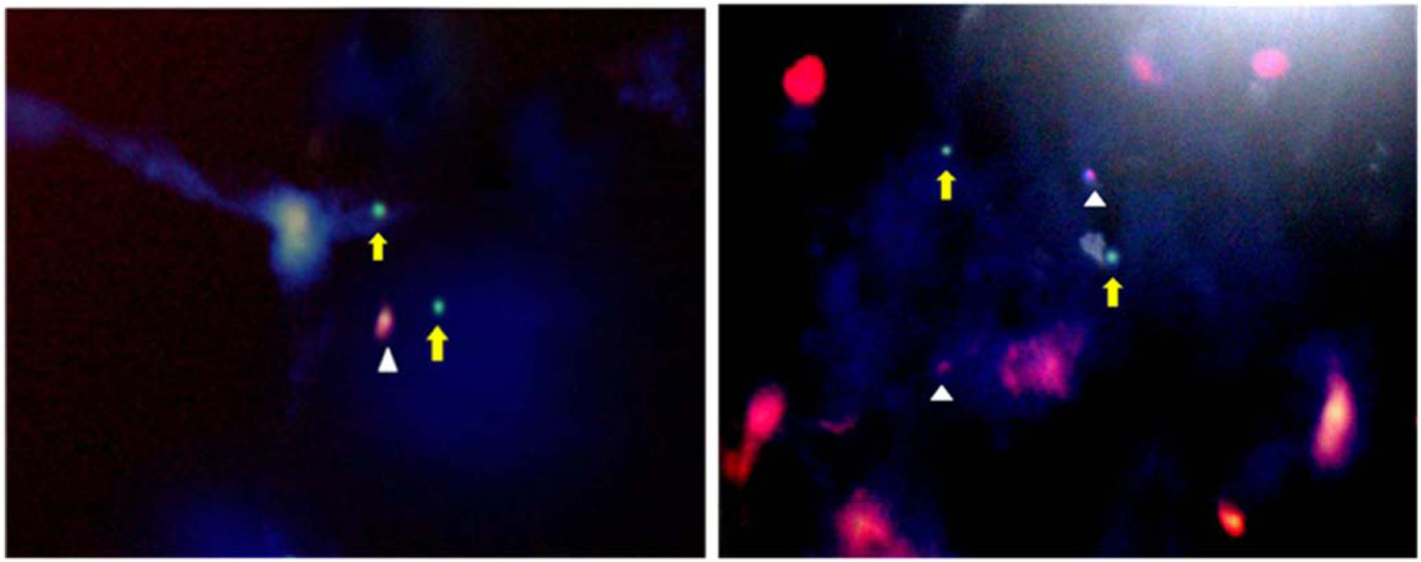
**FISH images of formalin fixed paraffin embedded sections of BAC.** Red signals (white arrow heads) are indicative of the p53 gene probe, while green signals (yellow block arrows) are indicative of centromeric probe of chromosome 17. The blue color is that of the DAPI counter stain. The presence of a single red signal per cell reflects the presence of a single copy of the p53 gene, i.e. p53 deletion (left), right photo represents normal cell.

One hundred cells were examined for each case. The cut offs were estimated by the clinical pathology laboratory by detection of the p53 gene locus deletion in 20 normal cases and calculating their mean and SD. Cases were considered positive if their score exceeded mean + SD of normal samples i.e. more than 6. Three samples (37.5%) were found to have a deletion of the p53 gene as evidenced by the presence of a single copy number of the gene (Figure 3).

#### Karyotyping technique for peripheral blood mononuclear cells

**Figure 4:**
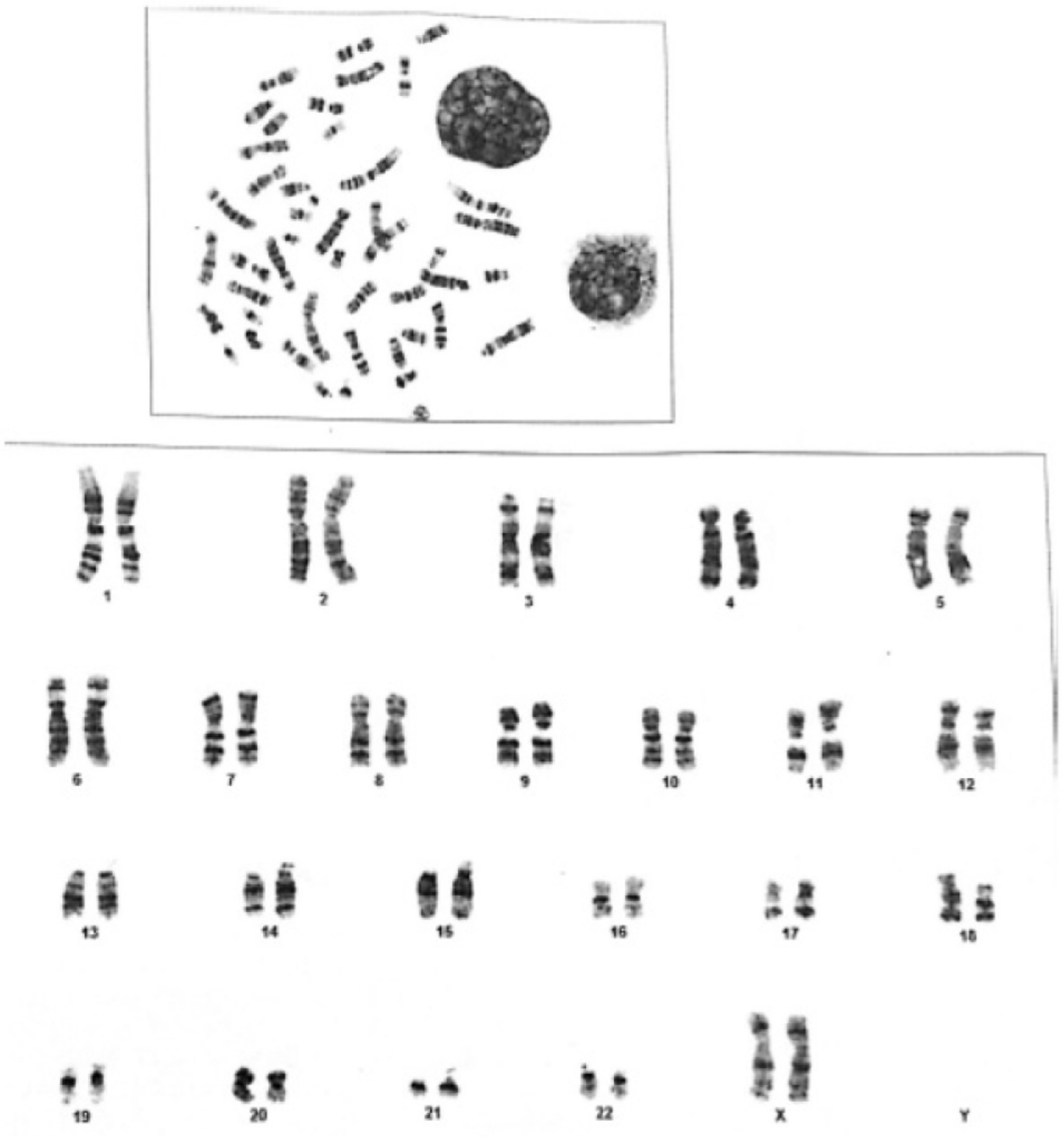
**Karyotyping of peripheral blood lymphocyte shows normal female 46xx chromosomes.**

**Figure 5:**
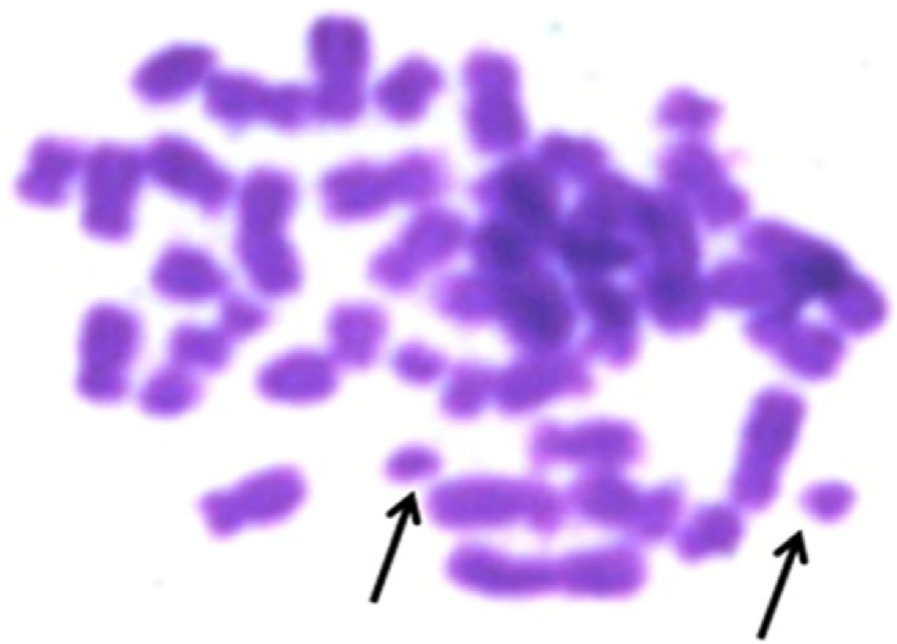
**Karyogram of peripheral blood mononuclear cell shows structural chromosomal abnormalities in the form of chromosome fragmentations with a normal male karyotype (46, XY).** Arrows point at fragments of chromosomes.

No numerical chromosomal aberrations were detected in peripheral blood mononuclear cells, where all samples showed a normal karyotype pattern (Figure 4). However, 3 cases showed evidence of chromosomal fragmentations (Figure 5).

## Discussion

The current study was conducted on 46 schistosomiasis *haemotobium* patients, 22 were diagnosed as active *S. haemotobium* infection and 24 were chronic cases complicated with bladder cancer. The mean age among patients of complicated chronic infection was 62.5 years approaches the mean age reported by [10] (54.3 years). A statistically significant difference (P = 0.0001) was found between the distribution of infection in Upper Egypt and Lower Egypt in both active and chronic infection groups where chronic infection was more prevalent (17/24) (70.38%) in Upper Egypt than active infection, which seemed to be more focused in Lower Egypt. Also, a significant statistical difference was found in the distribution in rural and urban areas (P = 0.004), where chronic infection was found to be more prevalent in rural areas (17/24) (70.38%). In the WHO report of 2011 (Report of an informal consultation on schistosomiasis control), the organization’s control measures of schistosomiasis in the Eastern Mediterranean region were considered to be successful, where Egypt was ranked as an area of low prevalence of infection and transmission in Egypt was expected to be interrupted. Therefore, the observed findings with acute cases (emerging active cases from Cairo & Giza) should direct the Egyptian researchers to maximize their efforts to explore extensively the reasons behind such change.

Cytogenetic analysis of parasitic diseases has been focusing on the studying of chronic complications, while active infection remains yet insufficiently explored. Available data about molecular genetic changes originate largely from animal studies as the study that was conducted by Ray et al. [11] who analyzed differential gene description after locally infecting laboratory animal with *Schistosoma* egg. In the present study, a trial was done to explore the chromosomal and cytokinetic genetic compromise in different schistosomiasis cases. The difference between the mean DNA content of cell nuclei in acute and chronic cases was found to be statistically significant, indicating a higher DNA content in samples of complicated chronic cases compared to active ones. Although the presence of diploid cells with high proliferation index in urethral cells of acute cases reflects the increased dynamics of the cell cycle in response to active infection. However, bladder tissue samples of chronic cases showed 5 samples with diploid nuclei, 6 samples were tetraploid while, 13 samples were aneuploid and all samples were with a high proliferative index which may reflect the role of chronic *S. haematobium* infection on exaggeration of cell cycle cytokinetics elucidating the role of DNA ploidy analysis as a predictor for malignant transformation in *S. haematobium* infected patients. Similarly, El-Hossainy et al*.* [12] have applied DNA morphometric analysis to classify bilharzial and non-bilharzial bladder tumors on the basis of the mean nuclear area (MNA) of malignant cells, however, they used Hematoxylin and Eosin instead of Feulgen stain used in our study.

The authors reported that the greater the amplification of the chromosome copy number (ploidy) in malignant cells, the greater was the mean nuclear area of these cells. This was associated with more progressive tumors with lymph node metastasis. However, there was no significant difference in the mean nuclear area of bilharzial and non bilharzial bladder tumors. These findings come in concordance with the present study, where the mean ± SD DNA content in tissue sections from BAC was calculated as 3.18 ± 0.65, while non-BAC samples had a mean DNA content of 2.54 ± 0.3. No significant statistical difference in DNA content was found between the two groups (P = 0.068).

Despite the fact that DNA ploidy analysis is not specific for bilharzial carcinoma, it could be used to follow up cases with chronic bilharzial infection to guard against occurrence of neoplastic changes, being safe, non-invasive, very simple and affordable. This hypothesis could be supported by Puntoni et al*.* [13] who found that DNA ploidy analysis was a great prognostic factor for superficial bladder tumors. In addition to its value in prognosis, DNA cytometry has proven to be a useful diagnostic technique for the evaluation of neoplastic transformation in various types of tissues by nuclear morphometry [14]. The DNA ploidy analysis was frequently used in the previous authors’ institution for the detection of DNA aneuploidy in diagnostically difficult or doubtful cases. The authors recommended its use over other procedures as Papanicolaou staining and Hematoxylin and Eosin staining for its accurate densitometric measurements. In the visible light spectrum, only the Feulgen reaction has been accepted as a stoichiometric procedure for exclusive staining of nuclear DNA in a reproducible, standardized manner [14] and the discussion about the appropriateness of other staining methods for diagnostic measurements appears to have come to an end [15,16].

Another cytogenetic tool for the detection of the geno-toxicity of *S. haematobium* is the FISH technique. Aly and Khaled [17] examined frozen tissue specimens obtained from 35 patients suffering from early-stage bilharzial bladder cancer. They performed the FISH technique to detect the most common chromosomal abnormalities in biopsies from these patients. They found that chromosomes number 9, 10, 17 and the Y chromosomes were affected either by gain or deletion. In an attempt to compare the pattern of chromosomal abnormalities in Egyptian bilharzial cancer bladder with Western cancer bladder, Shaw et al. [18] examined tumor samples from 70 schistosomiasis patients. The most common sites of allelic losses were the short arm of chromosome 9 followed by the short arm of chromosome 17 (17p) which carries the TP53 gene (one of the most important tumor suppressor genes, also called the guardian of the genome [18]. The long arm of chromosome 9 (9q), however, seemed to be affected in a limited number of patients. Its affection was rather a feature of the Western type of bladder cancer. Based on these findings, the authors suggested the importance of the genes on 9p and 17p in the genesis of bilharzial cancer bladder in comparison to the unimportant role of genes located on 9q. Di Cézar et al. [19] stated that alterations of chromosomes 7 and 9 are related to the initiation process; and of chromosome 17, to tumoral progression and recurrence.

Restricted by the limited financial support and lack of availability to study multiple gene loci related to BAC, it was chosen to focus on the detection of the p53 gene in this study. This choice was based on the documented importance of this gene in invasive BAC. Eight cases from the chronic infection group complicated with bladder cancer were selected and interphase FISH technique was performed on tissue samples using a locus specific probe targeting the p53 gene. Three cases (37.5%) showed a deletion of the p53 gene. The cancer of the 3 cases was reported to be of the invasive type.

The importance of the p53 gene in the etiology of cancer lies in the fact that it encodes for the p53 protein which is a nuclear phosphoprotein that acts as a gatekeeper at the G1-S checkpoint of the cell cycle which is responsible for the progression of the cell cycle and is crucial for controlling urothelial cell growth and maintaining genomic stability [8]. Zekri *et al*. [20] found that in 32.7% of 101 studied cases of bilharzial cancer bladder; there was a mutation in the p53 gene, suggesting a strong correlation between the p53 mutation and bladder carcinogenesis. P53 abnormalities are much more prevalent in invasive bladder cancer (>50%) compared to the non-invasive form, suggesting that loss of p53 plays a role in the development of the invasive potential of the tumor [21-24]. Osman et al. [25] have as well linked the presence of the p53 gene to lymph node metastasis and poor prognosis.

However, FISH technique has a major downfall; interphase and metaphase FISH can only detect known genetic aberrations, requiring the availability of the specific probe. Conclusively, FISH cannot serve as a screening test for chromosomal rearrangements since most FISH techniques can only detect selected known imbalances [24].

UroVysion™ is a multicolor fluorescence in situ hybridization assay which was evaluated by Bonberg et al. [26] to detect abnormalities in chromosomes 3, 7 and 17 and loss of the 9p21 locus in exfoliated urothelial cells. The authors concluded that the test is time-consuming and costly compared to voided urine cytology or other molecular markers for the early detection of bladder cancer. The authors also recommended the use of a simpler, faster and cheaper assay with a disease related locus specific single probe.

The study of peripheral blood lymphocytes in cancer patients has been a valuable research focus in an approach to identify increased risk of cancer development on the basis of detecting chromosomal abnormalities [27].

In Egypt, Khaled et al. [28] have conducted a study about the effect of *S. mansoni* infection on the chromosomal morphology of cultured peripheral blood lymphocytes of 24 schistosomiasis patients. They have found that *S. mansoni* was genotoxic to all 24 patients as evidenced by the presence of structural chromosomal abnormalities in the cultured cells. Among the chromosomal aberrations they detected, were chromosomal gaps, breaks and fragmentations. In the present study, cultured peripheral blood mononuclear cells from eight chronically infected patients were examined for chromosomal numerical aberrations as well as deletions or translocations that may imply malignant urothelial tissue transformation. Based on previous study by Shao et al., [18] who detected chromosome aberrations in chromosomes 9 and 11 in peripheral blood lymphocytes of bladder cancer cases, we hypothesized that such abnormalities could be detected in our cases. However, no numerical abnormalities were detected but noticeably three out of the eight cases showed chromosomal fragmentations that were detected in only 1or2cells per case. Moreover, we were not paying attention to elucidate other changes like gabs and breaks as they only indicate stress on the chromosomes by bilharzial toxins more than factual cancer transformation.

Chromosome fragmentation is a major form of mitotic cell death which is identifiable during common cytogenetic analysis by its unique phenotype of progressively degraded chromosomes [29]. Similarly, Halder and Fauzdar [30] performed karyotyping on only 3 patients and concluded that cytogenetic analysis by conventional chromosomal banding techniques (karyotyping), although an important standard method, requires cell culture, skilled personnel and is labor as well as time expensive. Nevertheless, karyotyping has played an important role in the understanding of human cancers since the 1970’s; its general application is still limited, since it is based on analyzing metaphase cells which are difficult to obtain in many solid tumors. Furthermore, the exact position of chromosome breakpoints roughly detected by karyotyping needs to be defined and confirmed by fluorescence in situ hybridization FISH analysis which augments the burden and cost to achieve diagnosis [31].

In general, the selection of the ideal assay depends on whether the goal is detection, screening, monitoring, surveillance, or prediction of the invasive or metastatic potential of the disease. Various cytogenetic techniques have been employed in the present study for the detection of genomic abnormalities associated with urinary schistosomiasis. Targeting of an efficient, feasible and affordable screening and follow up tool for the complications threatening high risk subjects will certainly be considered as a breakthrough in the control of urinary schistosomiasis. The current situation of this serious disease and its causative agent has to be further investigated in Egypt especially that it targets unfortunate school children, who are at a higher risk for infection and are more vulnerable to the development of neoplastic changes later in life due to the acquisition of infection at a young age as proved by Rambau et al. [10].

## Conclusions

In general, Genetic biomonitoring of population exposed to potential carcinogens as *S. haematobium* infection is an early warning system for genetic instability and neoplasm transformation especially in our area where control measures could still be implemented. The present study has been focusing on some cytogenetic techniques that help to disclose the impact of urinary bilharziasis on host genetics. DNA morphometry has been found to be of value in the detection of gross genetic changes. Though non-specific, it could be considered as a rough estimation of the chromosomal status. In addition, it is a valuable prognostic factor in established schistosomiasis *haematobium* induced malignancy. It has the great advantage of being applicable on urine cells, which offers a simple, non costly and non-invasive screening tool. This makes it suitable for the prediction of a tendency towards genetic instability in active *S. haematobium* patients. FISH technique is a sensitive technique that can determine specific gene loci. Yet, in invasive neoplasm, its value is limited. In addition to its high cost that makes this technique not feasible for screening of high risk patients. Karyotyping has been found of limited implication being non-specific, since it detects peripheral blood chromosomal changes. Also the karyotyping technique is tedious and time-consuming. The multiplicity of its steps including culture of peripheral blood mononuclear cells, preparation of slides for staining & interpretation of results increase the probability for technique failure.

## Methods

### Ethical approval

This study was conducted in compliance with the Helsinki Declaration and was approved by ethical committee of Faculty of Medicine, Cairo University. Written informed consents were obtained from all participants.

### Patients

The present work was carried out on 22 uncomplicated schistosomiasis *haematobium* patients attending the outpatient clinic of Theodor Bilharz Research Institute, Giza, Egypt and 24 complicated schistosomiasis *haematobium* inpatients which were admitted in the Urosurgery department at Kasr Al-Ainy Teaching hospital, Faculty of Medicine, Cairo University over the period from November 2012 till December 2013. The 22 active *Schistosoma haematobium* infections were diagnosed microscopically by observation of parasitic eggs in their urine samples. The 24 chronic complicated schistosomiasis *haemotobium* cases were diagnosed to have bladder cancer as evidenced by clinical (history of repeated cystitis with terminal haematuria), radiological (characteristic bilharzial changes in ultrasound and Computed Tomography scanning) and histopathological data. Patients with non bilharzial cancer bladder or those suffering from cancer in any other organs were excluded. Cases included in the present work were subjected to history taking, full clinical examination, complete urine analysis and parasitological examination. Feulgen stain quantitative nuclear morphometric analysis and computerized image analysis of stained slides were done for all tissue specimens and urine smears. Karyotying for peripheral blood mononuclear cells and FISH technique using locus specific probe targeting the p53 gene for tissue specimens were done for 8 chronic patients with cancer bladder.

### Sample collection

Urine specimens were collected from all cases included in this study. Fresh tumor tissue samples were obtained at the same time of surgical resection. A portion of each tumor specimen was fixed in 10% formalin. Tissue samples were taken from patients with previous biopsy reporting the presence of bladder cancer and *Schistosoma* egg pathology and before taking chemotherapy. Blood samples were collected from 8 chronically infected complicated cases in tightly sealed Vacutainer^©^ tubes coated with Sodium heparin. From each patient 6 cc of blood were withdrawn and divided into two blood collection tubes. The sample was then processed preferably immediately or within 2 hours.

### Parasitological examination of urine samples

Collected urine samples were subjected to direct microscopic examination using a light compound microscope at 100 × and 400 × magnifications, in addition to examination of the urine sediment after centrifugation at 3,000 rpm for 10 min. Urine samples were examined for the presence of *S. haematobium* eggs (140×70 μ) or other parasites using a nucleopore filtration device with a polycarbonate filter having a diameter of 13 mm and a pore size of 12 μm. Some of the urine samples were smeared on slides and fixed with absolute ethanol to be stored for Feulgen dye staining and subsequent whole human epithelial cells chromosomal cytophotometric analysis.

### Feulgen technique

Feulgen staining was done for all tissue specimens and urine smears. In addition, Formalin fixed paraffin embedded (FFPE) from 4 patients with non- Bilharzia associated bladder cancer (BAC) were examined as a comparative group. Tissue sections and urine smears were rehydrated in an ethanol series, 1 minute in each concentration. The slides were then rinsed briefly with cold 1 N hydrochloric acid and then placed in pre-warmed hydrochloric acid at 60 degrees for 10–30 minutes. Brief rinses were then carried out in cold HCl and in distilled water. The slides were then placed in Schiff’s reagent for 30–60 minutes at room temperature. This is followed by three sulfite rinses for 1 minute each and then the slides are washed well with water. Sulfite rinses are formed of 5 ml Potassium metabisolfite (10% aqueous solution) and 95 ml of 1 N HCl. After drying, cover slips are added to the slides using DPX mountant [32].

#### DNA analysis of feulgen stained slide preparations

Feulgen stained slides were analyzed by quantitative morphometry, by which morphology of the pink stained nuclei was analyzed for parameters such as shape, size, integrated optical density and nuclear area. The morphometric analysis was performed at the Pathology Department, National Research Center, using the Leica Qwin 500 Image Analyzer (LEICA Imaging Systems Ltd, Cambridge, England,) which consists of a Leica DM-LB microscope with a JVC color video camera attached to a computer system Leica Q 500IW. Feulgen stained slides were examined by a compound light microscope at 100× and 400× magnification. Morphometric measurements were taken from the real – time image transmitted from the microscope and visualized on the video monitor. The results appeared automatically on the monitor in the form of the diameter measured in (μm) and area in (μm^2^) with calculation of the mean and standard deviation. In addition, the minimum length and the maximum length of nuclei with abnormal morphology were measured. The morphological features of epithelial cell nuclei in the study samples were measured relative to reference diploid nuclei, the parameters of which were stored on the image analyzing software. Only separate, intact nuclei were measured. Distorted or overlapping nuclei and nuclear fragments were manually eliminated from measurements. The optical density of the selected nuclei in each microscopic field is then measured and automatically converted by the system into DNA content. Many fields were selected until the desired number of nuclei (from 100 – 150) has been measured. The results were displayed as a frequency histogram on the monitor generated by blotting the DNA content versus the number of nuclei counted according to [33].

### Karyotyping

Peripheral blood of 8 chronic complicated bilharzial patients with bladder cancer was examined for chromosomal abnormalities by examination of cultured peripheral blood mononuclear cells. It was conducted following a standard protocol with slight modifications. Half ml heparinized whole blood was cultured in RPMI with L-glutamine medium supplemented with 20% fetal bovine serum (FBS) (Eurolone, Europe), 200 ul phytohaemaglutinin, 100 ul penicillin and streptomycin, 100 ul antimycotic and 25 ul preserved heparin. Thymidine (100 μl) was added to the cell culture and the mixture was incubated at 37°C for 18 hours. 2- deoxycytidine (100 μl) was added to the mixture and incubation was done at 37°C for 2 hours. Then 100 μl of colcemid were added to achieve mitosis arrest and incubation was a done at 37°C for 30 min. A few drops of a hypotonic KCl solution were added to the culture and the whole mixture was then centrifuged at 1000 rpm for 10 minutes. The supernatant was then discarded and hypotonic KCl solution was added again in a gradual manner till the whole solution volume reached 10 ml. Fixation was done by adding a 3:1 methanol: acetic acid solution. The prepared slides were stained with Wright’s stain by covering the slides with the stain for 15 minutes. Before staining, the slides were treated with trypsin for 2–4 seconds to produce banding by protein digestion. After location of the metaphase spreads, the slides were examined under a 1000x magnification by a microscope connected to a computer with software (Genetix cytovision software number 7.0) that was able to identify and organize chromosomes into a karyogram. The cultured cells were examined for numerical chromosomal abnormalities as well as deletions or translocations that could indicate malignant transformation.

### FISH procedure

FFPE tissue sections of the 8 complicated bilharzial cases were prepared for FISH technique using the Vysis paraffin pretreatment kit II (Kit Order #32-80121, Abott molecular). Slides were immersed in xylene for 5–10 minutes at ambient temperature. Dehydration of the slides in 100% ethanol for 1 minute was done. Protease treatment was done according to manufacture instructions. Ten μl of the specific probe [Cytocell aquarius, p53, 17p13.1, red, 17c(D17Z1), green] was then dropped on the slide and incubation at 37°C for one day. The slides were then washed twice. Wash one was preheated to 72°C in a water bath. It was composed of 49 cc of distilled water, 1 cc of 20 x (SSC) and 3 drops of NP40. The slide was left in wash 1 for 2 minutes. Wash two was then done for several minutes. It was made of 45 cc of distilled water, 5 ml of 20 × SSC and 1 drop of NP40. After that, the DAPI counter stain was added to the slide. The slide was then ready for examination with a fluorescent microscope with triple band pass filter (DAPI/FITC/TexasRed) in order to identify the blue fluorescence of the DAPI counter stain, the green fluorescence of the control centromere probe and the red fluorescence of the locus specific probe.

## Statistical methods

Data was analyzed using IBM SPSS advanced statistics version 16 (SPSS Inc., Chicago, IL). Numerical data were expressed as mean and standard deviation or median and range as appropriate. Qualitative data were expressed as frequency and percentage. Chi-square test was used to examine the relation between qualitative variables. Comparison between two groups was done using Paired t test. A p-value ≤ 0.05 was considered significant.

## Abbreviations

FISH: Fluorescent in situ hybridization
BAC: Bilharzia associated bladder cancer
FBS: Fetal bovine serum
FFPE: Formalin fixed paraffin embedded
